# Dimerization of EGFR and HER2 induces breast cancer cell motility through STAT1-dependent ACTA2 induction

**DOI:** 10.18632/oncotarget.10843

**Published:** 2016-07-26

**Authors:** Myeongjin Jeon, Daeun You, Soo Youn Bae, Seok Won Kim, Seok Jin Nam, Hyeon Ho Kim, Sangmin Kim, Jeong Eon Lee

**Affiliations:** ^1^ Department of Surgery, Samsung Medical Center, Gangnam-gu, Seoul 06351, Korea; ^2^ Department of Health Sciences and Technology, SAIHST, Sungkyunkwan University, Gangnam-gu, Seoul 06351, Korea

**Keywords:** ACTA2, HER2, STAT1, invasion, metastasis

## Abstract

The dimerization of EGFR and HER2 is associated with poor prognosis such as induction of tumor growth and cell invasion compared to when EGFR remains as a homodimer. However, the mechanism for events after dimerization in breast cancer models is not clear. We found that expressions of alpha-smooth muscle actin (ACTA2) and signal transducer and activator of transcription 1 (STAT1) significantly increased with transient or stable overexpression of HER2 in EGFR-positive breast cancer cells. ACTA2 and STAT1 expression was also increased in HER2-positive breast cancer patients. In contrast, ACTA2 expression was decreased by HER2 siRNA. Next, we investigated the co-relation between STAT1 and ACTA2 expression. Basal ACTA2 expression was significantly decreased by treatment with the STAT1 inhibitor fludarabine or the JAK2 inhibitor AG490. In contrast, ACTA2 expression was increased by STAT1 overexpression. Levels of ACTA2, STAT1, and HER2 were increased and relapse free survival was decreased in high-risk breast cancer patients. We also investigated the effect of ACTA2 on cell motility, which was suppressed by ACTA2 shRNA overexpression in MDA-MB231 HER2 and 4T1 mammary carcinoma cells. The number of lung metastatic nodules was significantly decreased in ACTA2 knockdown mice. Taken together, these results demonstrated that induction of ACTA2 by EGFR and HER2 dimerization was regulated through a JAK2/STAT1 signaling pathway, and aberrant ACTA2 expression accelerated the invasiveness and metastasis of breast cancer cells.

## INTRODUCTION

The HER2 gene is amplified in approximately 25–30% of primary breast tumors and is one of the most significant genetic changes in breast cancer [1]. HER2-positive tumors are associated with increased cell proliferation, motility, tumor invasiveness, metastasis, angiogenesis, and decreased apoptosis [2, 3]. In addition, breast cancer patients with HER2 overexpression show aggressive clinical course, including poor disease-free and overall survival, chemo-resistance, and shorter time to relapse [4, 5]. HER2-targeted therapeutic agents such as trastuzumab, pertuzumab, and lapatinib have clinical efficacy [6]. However, despite the clinical benefits of these HER2-targeted therapies, the response rate for trastuzumab as a single agent remains less than satisfactory at around 35% and the vast majority of tumors that respond to trastuzumab develop resistance within 1 to 2 years of treatment [6].

HER2 has no known direct ligand. Its activation is achieved through dimerization with other ligand-bound HER family receptors such as EGFR [7, 8]. EGFR and HER2 are frequently overexpressed in breast cancer, and their dimerization is associated with more aggressive clinical behavior [7, 8]. The heterodimerization of EGFR with HER2 induces a more potent EGFR activation than EGFR homodimerization, resulting in hyperactivation of several downstream signaling pathways, including MAPK and PI-3K/Akt pathways that regulate cell proliferation, survival, migration, angiogenesis, and metastasis [7, 9–11]. Brabender et al. reported that lung cancer patients with EGFR and HER2 are associated with poor prognosis compared with patients expressing EGFR or HER2 alone [12]. Although the dimerization of HER2 and other receptor family contributes to aggressiveness of breast cancer, the mechanism for this observation is not well understood.

Alpha-smooth muscle actin (ACTA2) contributes to cell-generated mechanical tension and maintenance of cell shape. ACTA2 is also pivotal in tumor cell migration and invasion [13]. Increased ACTA2 expression mediates the epithelial-mesenchymal transition (EMT) that is implicated in tumor progression and metastasis during cancer development in lung and pancreatic cancers [14, 15]. Colorectal cancer patients with high ACTA2 expression have a shorter disease-free survival and ACTA2 expression is a significant prognostic factor comparable to lymph node metastasis [16, 17]. ACTA2 expression is regulated by various stimuli. IL-1β- and IL-6-induced ACTA2 expression is attenuated by anti-TGF-β1 neutralizing antibody [18]. Retinol and its metabolites such as all-trans retinoic acid and 9-retinoic acid inhibit ACTA2 expression in pancreatic stellate cells [19]. TGF-β1-induced ACTA2 expression is mediated through PI-3K and p38 MAPK-dependent pathway [20].

The main aim of this study was to assess the mechanism between the dimerization of EGFR and HER2 and motility of breast cancer. HER2 overexpression resulted in enhancement of ACTA2 and STAT1 expression in EGFR-positive breast cancer cells. In addition, levels of ACTA2 and STAT1 expression were highly expressed in HER2-positive breast cancers. ACTA2 expression was increased by STAT1 overexpression. Induction of ACTA2 by HER2 overexpression was decreased by blockage of the JAK2/STAT1 signaling pathway. Finally, ACTA2 silencing completely suppressed cell migration and invasion in HER2-overexpressing breast cancer cells. Lung metastasis of 4T1 mammary carcinoma cells was also suppressed by ACTA2 silencing in an *in vivo* mouse model. Taken together, these results demonstrated that dimerization of EGFR and HER2 activates the JAK2/STAT1 signaling axis and induces ACTA2 expression in breast cancer cells. Furthermore, elevated ACTA2 triggered the motility of breast cancer cells.

## RESULTS

### ACTA2 and STAT1 expression are increased by HER2 overexpression in breast cancer cells

**Figure 1:**
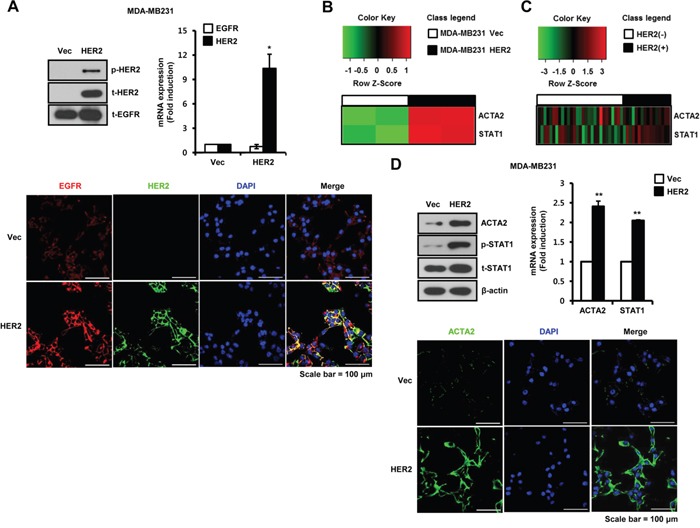
ACTA2 and STAT1 expression are increased by HER2 overexpression in breast cancer cells **A**. The levels of p-HER2 (pT877), t-HER2, and t-EGFR expression were analyzed by western blots. ACTA2 and STAT1 mRNA expression were analyzed by real-time PCR. EGFR and HER2 expression were analyzed by confocal microscopy. **B**. ACTA2 and STAT1 expression patterns were analyzed by cDNA microarray analysis. **C**. Heatmap of ACTA2 and STAT1 expression in tumors from breast cancer patients generated using R statistical software. **D**. ACTA2, p-STAT1 (pS727), t-STAT1, and β-actin expression analyzed by western blots. ACTA2 and STAT1 mRNA expression were analyzed by real-time PCR. ACTA2 expression was analyzed by confocal microscopy. Results are representative of three independent experiments. Values are means ± SEM. * *p* < 0.05, ** *p* < 0.01 vs. Vec. Vec; empty vector.

We investigated the relationship between EGFR and/or HER2 dimerization and breast cancer aggressiveness. Generally, MDA-MB231 cells highly express endogenous EGFR but not HER2 (Figure 1A). We established HER2-overexpressing MDA-MB231 breast cancer cells. We confirmed EGFR and HER2 expression in established cell models by western blots, real-time PCR, and immunofluorescence (Figure 1A). Colocalization of EGFR (red) and HER2 (green) on plasma membrane of HER2-overexpressing cells was represented in yellow (merge between red and green) (Figure 1A). Using vec-alone and HER2-overexpressing MDA-MB231 cells, we analyzed gene expression patterns using cDNA microarrays. Basal levels of ACTA2 and STAT1 expression were significantly higher in HER2 overexpressed cells than in vec-alone cells (Figure 1B). We also analyzed the patterns of ACTA2 and STAT1 expression in HER2-negative and HER2-positive breast cancers from patients using the GEO database (GSE19615). By analyzing the public dataset, we found that ACTA2 and STAT1 expression were relatively higher in HER2-positive than in HER2-negative breast cancers (Figure 1C). Additionally, we analyzed the expression patterns of other myoepithelial breast cells and EMT markers in HER2-negative and HER2-positive breast cancer patients using the GEO database (GSE19615). We verified that the expression levels of KRT14, CDH1, CDH2, VIM, SNAI1, SNAI2, TWIST1, and ZEB1 did not show a difference between HER2-negative and HER2-positive breast cancer patients (Supplementary Figure S1A). Consistent with these results, we observed that ACTA2 expression and STAT1 phosphorylation increased in HER2-overexpressing MDA-MB231 cells (Figure 1D). ACTA2 mRNA increased to 2.41 ± 0.14-fold and STAT1 mRNA increased 2.05 ± 0.82-fold compared to vec-alone in HER2-overexpressing MDA-MB231 cells (Figure 1D). We also observed ACTA2 expression in vec-alone and HER2-overexpressing cells by immunofluorescence (Figure 1D). In addition, we confirmed the EGF-induced phosphorylation of signaling molecules in both vec-alone and HER2-overexpressing MDA-MB231 cells (Supplementary Figure S1B). Our results showed that STAT1 and STAT3 phosphorylation were increased in HER2-overexpressing MDA-MB231 cells (Supplementary Figure S1B). The levels of STAT1 and STAT3 phosphorylation were also additively increased by EGF treatment in HER2-overexpressing MDA-MB231 cells (Supplementary Figure S1B). However, our results showed that Erk and Akt phosphorylation did not change in HER2-overexpressing MDA-MB231 cells when compared with vector alone cells (Supplementary Figure S1B).

**Figure 2:**
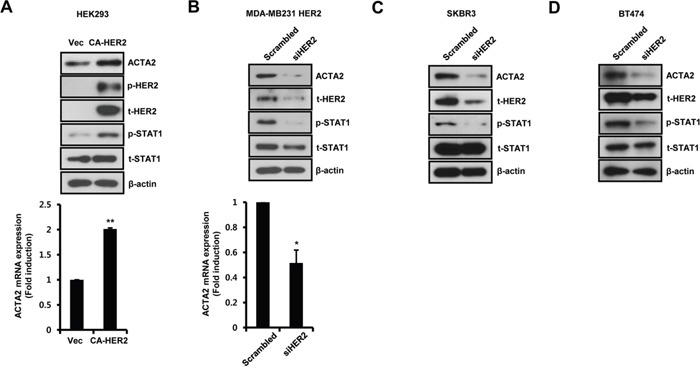
Alteration of HER2 expression regulates ACTA2 expression **A**. HEK293 cells were transiently transfected with vec or CA-HER2 for 48 h. **B-D**. HER2-overexpressing MDA-MB231, SKBR3, and BT474 cells were transiently transfected with scrambled or siHER2 for 48 h. The levels of ACTA2, p-HER2 (pT877), t-HER2, p-STAT1 (pS727), t-STAT1, and β-actin were analyzed by western blots. ACTA2 mRNA was analyzed by real-time PCR. Results are representative of three independent experiments. Values are means ± SEM. * *p* < 0.05, ** *p* < 0.01 vs. Vec or scrambled. Vec; empty vector.

We also investigated the transient effects of HER2 on ACTA2 and STAT1 expression. Constitutively active HER2 (CA-HER2) was transiently transfected into HEK293 cells for 48 h and samples were harvested for detection of mRNA and protein. ACTA2 protein and STAT1 phosphorylation increased with CA-HER2 overexpression (Figure 2A). Under the same conditions, ACTA2 mRNA increased to 2.01 ± 0.02-fold of the control level in CA-HER2-overexpressing cells (Figure 2A). We also investigated whether HER2 siRNA overexpression suppressed ACTA2 expression and STAT1 phosphorylation in HER2-overexpressing MDA-MB231 cells. ACTA2 protein and STAT1 phosphorylation decreased with HER2 siRNA overexpression (Figure 2B). ACTA2 mRNA also decreased to 0.52 ± 0.10-fold of the level in cells overexpressing scrambled HER2 siRNA (Figure 2B). In addition, we observed that ACTA2 protein and STAT1 phosphorylation decreased with HER2 siRNA overexpression in endogenously EGFR and HER2 expressed SKBR3 and BT474 cells (Figure 2C and 2D). These results indicated that ACTA2 expression and STAT1 activity were regulated by HER2 levels in breast cancer cells.

### Basal ACTA2 expression is decreased by the blockage of JAK2/STAT1 pathway

**Figure 3:**
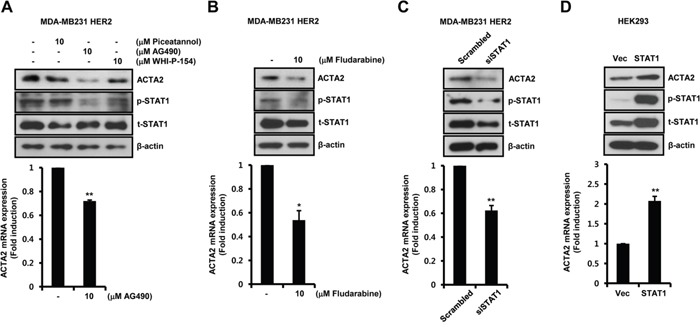
Basal ACTA2 expression is decreased by the blockage of JAK2/STAT1 pathway **A, B**. After serum starvation for 24 h, HER2-overexpressing MDA-MB231 cells were treated with 10 μM piceatannol, AG490, WHI-P-154, or fludarabine, for 24 h. **C**. HER2-overexpressing MDA-MB231 cells were transiently transfected with scrambled or siSTAT1 for 48 h. **D**. HEK293 cells were transiently transfected with vec or STAT1 for 48 h. ACTA2, p-STAT1 (pS727), t-STAT1, and β-actin expression was analyzed by western blots. ACTA2 mRNA was analyzed by real-time PCR. Results are representative of three independent experiments. Values are means ± SEM. * *p* < 0.05, ** *p* < 0.01 vs. (-) control, scrambled, or Vec. Vec; empty vector.

To investigate the regulatory mechanism of ACTA2 expression by HER2 overexpression, we examined the effect of specific inhibitors against STAT1 upstream signaling molecules. Basal levels of ACTA2 mRNA and protein decreased in HER2-overexpressing MDA-MB231 cells with the specific JAK2 inhibitor AG490, but not with the specific JAK1 inhibitor piceatannol or the specific JAK3 inhibitor WHI-P-154 (Figure 3A). Levels of ACTA2 mRNA after AG490 treatment decreased to 0.72 ± 0.01-fold the control level in HER2-overexpressing MDA-MB231 cells (Figure 3A). STAT1 phosphorylation was also completely suppressed by AG490 (Figure 3A).

To examine the effect of fludarabine, a specific STAT1 inhibitor, on HER2-overexpressing MDA-MB231 cells, we treated cells with fludarabine for 24 h. ACTA2 protein was significantly decreased by fludarabine (Figure 3B). Under the same conditions, ACTA2 mRNA decreased to 0.54 ± 0.08-fold of the control level (Figure 3B). STAT1 phosphorylation was suppressed by fludarabine (Figure 3B).

We investigated if STAT1 directly regulated ACTA2 expression. We examined whether STAT1 siRNA overexpression suppressed ACTA2 expression in HER2-overexpressing MDA-MB231 cells. ACTA2 protein decreased with STAT1 siRNA overexpression (Figure 3C). ACTA2 mRNA also decreased to 0.62 ± 0.04-fold of the level in cells overexpressing scrambled STAT1 siRNA (Figure 3C). In addition, HEK293 cells were transfected with STAT1 gene. Levels of ACTA2 protein and mRNA were increased with STAT1 overexpression (Figure 3D). The level of ACTA2 mRNA increased by 2.07 ± 0.12-fold of the vec level in STAT1-overexpressing cells (Figure 3D). We confirmed the levels of phospho-STAT1 and total-STAT1 expression in HEK293 cells (Figure 3D). Based on the results, dimerization of EGFR and HER2 triggered JAK2/STAT1 signaling pathway and induced ACTA2 expression.

### Clinical significance of ACTA2, STAT1, and HER2 expression in breast cancer patients

**Figure 4:**
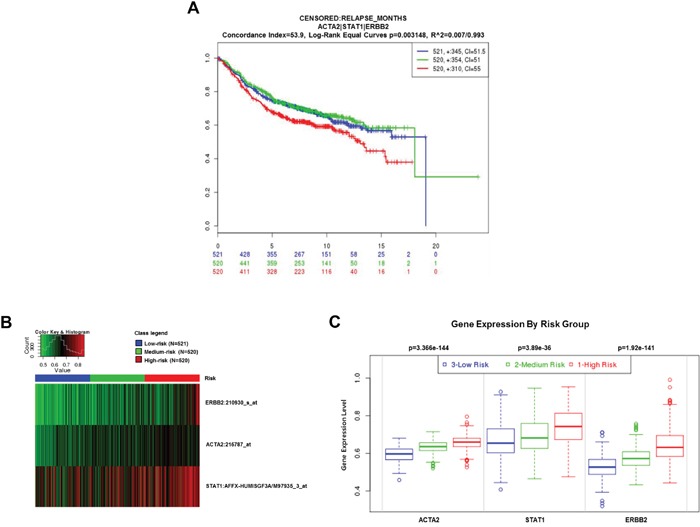
Clinical significance of ACTA2, STAT1, and HER2 expression in breast cancer patients The clinical value of ACTA2, STAT1, and HER2 was analyzed by SurvExpress software (http://bioinformatica.mty.itesm.mx/SurvExpress). **A**. Kaplan-Meier curves (relapse-free survival). **B**. Heatmap. **C**. Box plots.

To evaluate the clinical significance of ACTA2, STAT1, and HER2 expression in breast cancer patients, we performed an analysis in SurvExpress (breast cancer recurrence data, 9 datasets from 7 authors) using an average for genes with multiple probesets, three risk groups determined by prognostic index median, and Cox fitting. Results are shown as Kaplan-Meier curves for risk groups, a heatmap of ACTA2, STAT1, and HER2 expression, and box plots of ACTA2, STAT1, and HER2 expression by gene and risk group (Figure 4). High-risk breast cancer patients with highly expressed ACTA2, STAT1, and HER2 had significantly decreased relapse-free survival (Figure 4A). In addition, ACTA2, STAT1, and HER2 expression were significantly higher in the high-risk group than the low-risk group (Figure 4B and 4C). This result suggested that aberrant ACTA2, STAT1, and HER2 expression were associated with poor clinical outcomes in breast cancer patients.

### ACTA2 silencing decreases breast cancer cell motility

**Figure 5:**
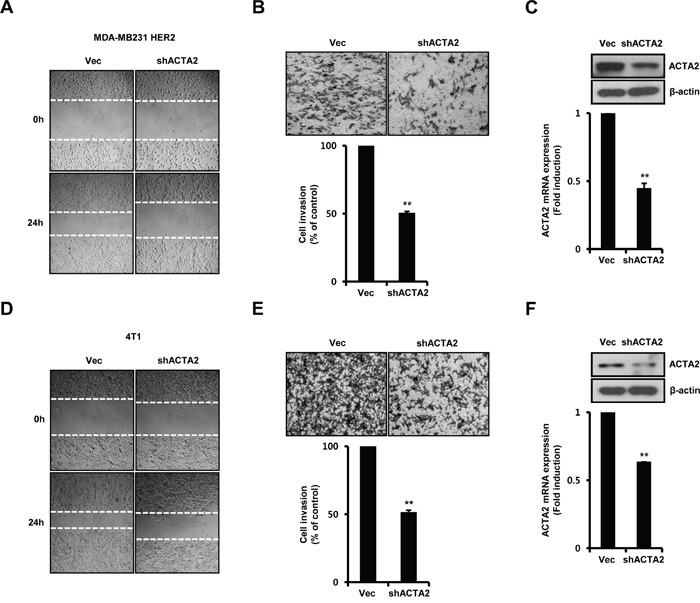
ACTA2 silencing decreases breast cancer cell motility MDA-MB231 HER2 **A-C**. and 4T1 mammary carcinoma cells **D-F**. were stably transfected with vec or ACTA2 shRNA for 48 h and selected with puromycin. (A, D) Migrating ability of cells overexpressing scrambled or ACTA2 shRNA analyzed by wound healing assays. (B, E) Invasiveness of cells with vec or ACTA2 shRNA was analyzed by Boyden chamber assays. (C, F) ACTA2 and β-actin expression was analyzed by western blots. ACTA2 mRNA was analyzed by real-time PCR. Results are representative of three independent experiments. Values are means ± SEM. ** *p* < 0.01 vs. Vec. Vec; empty vector.

We investigated the effect of ACTA2 on breast cancer cell motility. HER2-overexpressing MDA-MB231 cells and 4T1 mammary carcinoma cells were stably transfected with vec-alone or ACTA2 shRNA. Migration of HER2-overexpressing MDA-MB231 (Figure 5A) and 4T1 mammary carcinoma cells (Figure 5D) significantly decreased with ACTA2 shRNA overexpression. Invasiveness of HER2-overexpressing MDA-MB231 (Figure 5B) and 4T1 mammary carcinoma cells (Figure 5E) was also suppressed by ACTA2 shRNA overexpression. Rates of cell invasion by ACTA2 silencing decreased to 50.59% (HER2 overexpressed MDA-MB231 cells) and 51.67% (4T1 mammary carcinoma cells) of the vec-alone cells (Figure 5B and 5E). We verified that the levels of ACTA2 protein and mRNA decreased with ACTA2 shRNA overexpression in MDA-MB231 HER2 and 4T1 cells (Figure 5C and 5F). ACTA2 mRNA expression decreased to 0.45 ± 0.03-fold (HER2-overexpressing MDA-MB231 cells) and 0.64-fold (4T1 mammary carcinoma cells) of controls with ACTA2 shRNA overexpression (Figure 5C and 5F). Based on these results, we propose that ACTA2 induction by HER2 overexpression was involved in breast cancer cell motility.

### ACTA2 silencing decreases the metastatic potential of breast cancer cells

**Figure 6:**
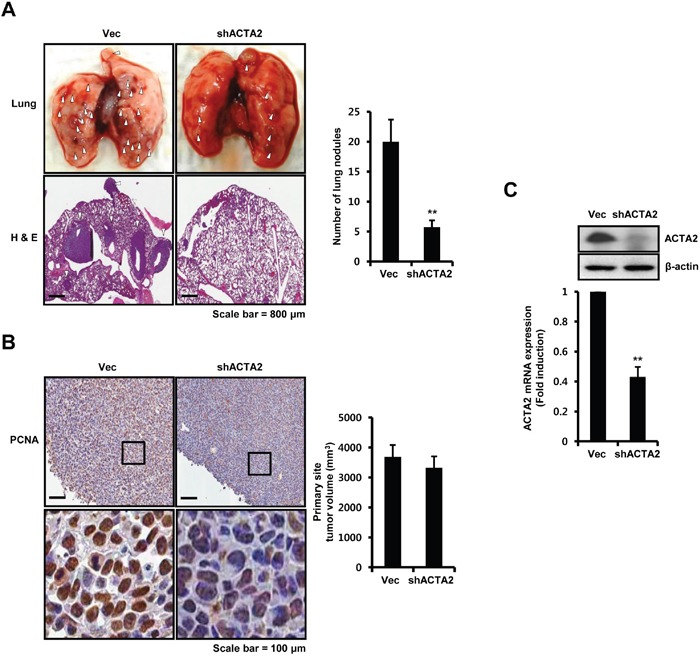
ACTA2 silencing decreases the metastatic potential of breast cancer cells Secondary mammary fat pads of mice were injected with 4T1 mammary carcinoma cells overexpressing vec or ACTA2 shRNA. **A**. After 24 days, lung tissues were removed, divided into vec (n = 5) or ACTA2-knockdown (n = 5) groups and analyzed for metastatic events. White arrow head, metastatic nodules. The number of metastatic nodules was counted in lung tissues. **B**. PCNA expression and tumor volume were measured to determine tumor growth. **C**. The levels of ACTA2 and β-actin expression were analyzed by western blots. ACTA2 mRNA was analyzed by real-time PCR. Values are means ± SEM. ** *p* < 0.01 vs. Vec.

We investigated the effect of ACTA2 on metastasis using 4T1 mammary carcinoma cells, which are highly tumorigenic and a model of invasion. We injected vec- or ACTA2 shRNA-transfected 4T1 mammary carcinoma cells into right secondary mammary fat. The metastatic potential of 4T1 mammary carcinoma cells xenograft tumors was suppressed by ACTA2 shRNA overexpression (Figure 6A). The number of lung metastatic nodules was decreased in ACTA2 knockdown mice (5.75 ± 1.12 nodules) compared with vec-alone mice (20 ± 3.67 nodules) (Figure 6A). In addition, we performed histological analysis of lungs using H&E staining. Metastatic sites were significantly increased in vec-alone mice compared with shACTA2 mice (Figure 6A). Although final tumor volume was not distinctly different between the vec-alone and ACTA2 shRNA-overexpressing mice, expression of PCNA was decreased in ACTA2-knockdown mouse tumors (Figure 6B). Under the same conditions, ACTA2 protein and mRNA decreased in ACTA2-knockdown mouse tumors compared with control mouse tumors (Figure 6C). ACTA2 mRNA decreased to 0.43 ± 0.07-fold of controls in ACTA2-knockdown mouse tumors (Figure 6C). We also examined the levels of ACTA2 protein expression in metastatic lung tissues (Supplementary Figure S2). As shown in Supplementary Figure S2, we observed that ACTA2 protein expression significantly decreased in ACTA2-knockdown lung metastatic tumors when compared with control lung metastatic tumors. In addition to this, we observed that COX2 expression was not associated with ACTA2, STAT1, and HER2 expression in breast cancer patients and to the clinical outcomes (Supplementary Figure S3). Therefore, we propose that elevated ACTA2 expression induced the metastatic and proliferative potential of breast cancer.

## DISCUSSION

HER2 is overexpressed in approximately 25–30% of breast cancer and prefers other HER family receptors as heterodimerization partners [4, 7, 8]. EGFR and HER2 double positive patients significantly increase recurrence rates compared with patients expressing EGFR or HER2 in non-small cell lung cancer [12]. EGFR overexpression is associated with prognostic and predictive value in HER2-positive breast cancer patients [21]. In addition, HER2 and HER3 heterodimers are correlated with decreased relapse-free and overall survival of breast cancer patients [22]. The dimerization of HER3 and HER2 synergistically increases heregulin-β1-induced MMP-1 and MMP-9 expression in breast cancer cells [23]. Consistent with these reports, our results showed that expression of the cell motility-related gene ACTA2 was significantly increased in HER2-overexpressing cells compared with vec-alone cells. Therefore, we demonstrated that heterodimerization of EGFR and HER2 contributes more to the aggressiveness of breast cancer than EGFR homodimerization.

We also recently reported that induction of fibronectin by HER2 overexpression increases adhesion and invasion of breast cancer cells [24]. Hartman et al. reported that HER2 overexpression triggers a pro-inflammatory IL-6 autocrine signaling loop required for HER2-mediated tumorigenesis in breast cancer [25]. In addition, HER2-induced TGF-β production leads to breast cancer metastasis through activation of smad3 [26]. We found that HER2 overexpression increases levels of ACTA2 and STAT1 in breast cancer cells. ACTA2 and STAT1 expression was also higher in HER2-positive breast cancers than in HER2-negative cancers. Based on these results, we propose that increased ACTA2 expression is important for cell invasion and migration in EGFR and HER2-positive breast cancer cell models. We investigated the regulatory mechanism of ACTA2 expression in EGFR-positive and/or HER2-positive breast cancer cells.

When ligands such as EGF and heregulin bind HER-family receptors, they activate intracellular signaling, leading to cell division, motility, survival and angiogenesis [27]. Kim et al. reported that phosphorylation of JNK, Akt, and Erk by heregulin-β1 is significantly increased in HER3 and HER2-positive breast cancer cells [23]. In this study, we also found that the level of STAT1 mRNA and the activity of STAT1 are higher in cells positive for EGFR and HER2 cells than in EGFR-positive cells. ACTA2 expression is regulated by several stimuli, such as TGF-β1, sphingosine-1-phosphate, and TNF-α [26, 28, 29]. TGF-β1 stimulates induction of ACTA2 and fibronectin via upregulation of snail and the activation of smad3 during EMT [26, 28]. Here, we found for the first time that the level of ACTA2 mRNA and protein in HER2-overexpressing cells decreased with the specific JAK2 inhibitor AG490 or the STAT1 inhibitor fludarabine. In contrast, basal ACTA2 expression increased with STAT1 overexpression. Therefore, we demonstrated that induction of ACTA2 by HER2 overexpression was mediated through a JAK2/STAT1-dependent signaling pathway.

Aberrant ACTA2 expression is associated with poor clinical outcomes in lung and pancreatic cancers [14, 15]. ACTA2 silencing significantly increases E-cadherin expression but decreases vimentin expression in lung adenocarcinoma cells [14]. TGF-β1-mediated ACTA2 triggers tumor invasion during the EMT [30, 31]. However, the prognostic significance of ACTA2 expression in breast cancer patients has not been fully elucidated. We found that breast cancer patients with tumors with high ACTA2 and HER2 levels had a poorer prognosis than patients with tumors with low ACTA2 and HER2 levels. Furthermore, our results showed that downregulation of ACTA2 by shRNA suppressed cell migration and invasion in HER2-overexpressing MDA-MB231 cells. ACTA2 silencing also decreased the metastatic potential of breast cancers. These findings indicated that induction of ACTA2 by HER2 was directly or indirectly involved in the survival of breast cancer patients through the regulation of metastatic potential.

The aim of this study was to clarify the mechanism of breast cancer motility by EGFR and/or HER2 dimerization. The levels of ACTA2 and STAT1 were increased in HER2-overexpressing breast cancer cells and tumors from HER2-positive breast cancer patients. Aberrant ACTA2 and STAT1 expression correlated with worse clinical outcomes of breast cancer patients. This is new evidence about the regulatory mechanism of ACTA2 expression in HER2-overexpressing cells. In HER2-overexpressing cells, basal ACTA2 expression was completely decreased by fludarabine or AG490 treatment. In contrast, ACTA2 expression was increased by STAT1 overexpression. We also found that motility of breast cancer cells was suppressed by ACTA2 silencing in breast cancer in *in vitro* and *in vivo* models. In conclusion, we demonstrated that dimerization of EGFR and HER2 induced ACTA2 expression through a JAK2/STAT1-dependent signaling pathway that triggers breast cancer cell motility.

## MATERIALS AND METHODS

### Reagents

Cell culture media and antibiotics were from Life Technologies (Rockville, MD, USA). Fetal bovine serum (FBS) was from Hyclone (Logan, UT, USA). Rabbit monoclonal anti-phospho-HER2 (pT877), STAT1 (pS727), STAT3 (pY705), Erk1/2 (pT202/pT185), Akt1 (pS473), total-HER2, EGFR, and STAT1 antibodies were from Epitomics (Burlingame, CA, USA). ACTA2 and PCNA mouse polyclonal antibodies were from Abcam (Cambridge, UK). β-actin antibody was from Abfrontier (Seoul, Korea). Secondary horseradish peroxidase (HRP)-conjugated antibodies were from Santa Cruz Biotechnology (Santa Cruz, CA, USA). Fludarabine was from Selleckchem (Houston, TX, USA). Piceatannol and AG490 were from TOCRIS (Ellisville, MO, USA). WHI-P-154 was from MERCK (Darmstadt, Germany).

### Cell cultures and drug treatment

Empty vector (retroviral pBMN) or HER2-overexpressing MDA-MB231, wild type MDA-MB231 human breast cancer cells, and HEK293 cells were cultured in Dulbecco's modified Eagle's medium (DMEM) supplemented with 10% FBS, 100 IU/ml penicillin, and 100 μg/ml streptomycin. BT474, SKBR3, and 4T1 mammary carcinoma cells were cultured in RPMI1640 supplemented with 10% FBS, 100 IU/ml penicillin, and 100 μg/ml streptomycin. Cells were grown in a humidified atmosphere with 5% CO_2_ at 37°C. For drug-treatment experiments, after serum starvation for 24 h, cells were treated with indicated inhibitors for 24 h.

### Western blots

Cell lysates were prepared for detection of p-HER2, t-HER2, t-EGFR, ACTA2, p-STAT1, t-STAT1, p-STAT3, p-Erk1/2, p-Akt1, and β-actin. Equal amounts of protein (50 μg) were boiled for 5 min in Laemmli sample buffer and separated by electrophoresis using 10% sodium dodecyl sulfate polyacrylamide gels. Separated proteins were transferred to polyvinylidene fluoride membranes that were blocked with 10% skim milk in Tris-buffered saline (TBS) containing 0.01% Tween-20 (TBS/T) for 15 min. Blots were washed 3 times in TBS/T and incubated with anti-p-HER2, t-HER2, t-EGFR, ACTA2, p-STAT1, t-STAT1, p-STAT3, p-Erk1/2, p-Akt1, or β-actin in TBS/T buffer at 4°C overnight. Blots were washed 3 times in TBS/T and incubated with secondary HRP-conjugated antibodies (1:2,000 dilution) in TBS/T buffer. After 1 h at room temperature (RT), blots were washed 3 times in TBS/T. ECL™ prime reagent (GE Healthcare, Bucks, UK) was used for development.

### Real-time polymerase chain reaction

Total RNA was extracted from cells using TRIzol reagent (Invitrogen, Carlsbad, CA, USA), according to the manufacturer's protocol. Isolated RNA samples were used for RT-PCR. Total RNA (1 μg) was reverse transcribed into cDNA in 20 μl reactions using a first-strand cDNA synthesis kit for RT-PCR, according to the manufacturer's instructions (MBI Fermentas, Hanover, MD, USA). Gene expression was quantified by real-time PCR using a SensiMix SYBR kit (Bioline Ltd., London, UK) and 100 ng cDNA per reaction. Specific primer sets were used to detect mRNA (Table 1). An annealing temperature of 60°C was used for all primers. PCR was performed in a standard 384-well plate format with an ABI 7900HT real-time PCR detection system (Foster City, CA, USA). For data analysis, raw threshold cycle (C_*T*_) values were normalized to a housekeeping gene to obtain ΔC_*T*_. Normalized ΔC_*T*_ was calibrated to the control cell samples for ΔΔC_*T*_.

**Table 1:** Specific primer sequences for analysis of EGFR, HER2, ACTA2, STAT1, and β-actin mRNA expression

Gene name	Forward	Reverse
**EGFR**	AGC AAC AAC CCT GCC CTG TGC AAC	TTC AAG ACC TGG CCC AGT GCA TCC
**HER2**	CAC TTC AAC CAC AGT GGC AT	ATT CAC ATA CTC CCT GGG GA
**ACTA2**	AGA CAT CAG GGG GTG ATG GT	CAT GGC TGG GAC ATT GAA AG
**STAT1**	CAT GCG GTT GAA CCC TAC AC	ATT GGC TCT GGT GCT TCC TT
**β-actin**	TCA CCA TTG GCA ATG AGC GGT T	AGT TTC GTG GAT GCC ACA GGA CT

### Confocal microscopy

Vec and HER2-overexpressing MDA-MB231 breast cancer cells grown on four-well Lab-Tek chamber slides were allowed to adhere overnight, fixed for 20 min in 4% paraformaldehyde, and incubated at 4°C overnight with anti-EGFR (Cell Signaling Technology, Beverly, MA, USA), HER2 (Santa Cruz Biotechnology, Santa Cruz, CA, USA), or ACTA2 (Abcam, Cambridge, MA, USA). Cells were washed three times in phosphate buffered saline (PBS) and slides were incubated with AlexaFluor 594-conjugated goat anti-rabbit secondary antibody, AlexaFluor 488-conjugated goat anti-mouse secondary antibody, or AlexaFluor 488-conjugated goat anti-rabbit secondary antibody (1:250 dilution), respectively, for 60 min at RT. Cells were washed and slides were mounted in Vectashield H-1200/DAPI mounting media (Vector Laboratories, Burlingame, CA, USA). Confocal images were analyzed using a LSM780 confocal laser-scanning microscope (Carl Zeiss, Zena, Germany).

### Plasmid DNA transfection

Empty vector and constitutively active HER2 (CA-HER2) were from Addgene (Cambridge, MA, USA). The STAT1 plasmid was a generous gift from Dr. Ye Sang-Kyu (Seoul National University, Korea). Scrambled, HER2, and STAT1 siRNA were from Bioneer (Daejeon, Korea). Empty and ACTA2 shRNA were from Santa Cruz Biotechnology (Santa Cruz, CA, USA). Cells were seeded in 6-well plates. Transfection used Lipofectamine 2000 (Invitrogen) according to the manufacturer's protocol. Cells were maintained in culture media with Lipofectamine 2000 but without FBS and antibiotics for 24 h, then incubated in fresh culture media with 10% FBS for 24 h. Stably transfected cells were selected with a working puromycin concentration of 1–10 μg/ml.

### Analysis of public databases

Expression data were downloaded from the GEO database (GSE19615, http://www.ncbi.nlm.nih.gov/geo). Heatmaps of gene expression for HER2-negative and HER2-positive breast cancer patients were generated using R statistical software (R Foundation, Vienna, Austria). Prognostic importance of ACTA2, STAT1, and HER2 expression in breast cancers was evaluated using SurvExpress software (http://bioinformatica.mty.itesm.mx/SurvExpress).

### Wound healing assays

Empty and ACTA2 shRNA-transfected cells were seeded in a 6-well plate and cultured for 24 h. Cells were maintained in culture media without FBS for 24 h. Monolayers were scratched with a 200-μl pipette tip to create wounds and cells were washed twice with PBS to remove suspended cells. Cells migrating from the leading edge were photographed at 0 and 24 h using a CK40 inverted microscope (Olympus, Tokyo, Japan).

### Boyden chamber assays

The 24-well Boyden chambers with matrigel-coated filters (8-μm pore size) were from Becton-Dickinson (San Diego, CA, USA). Control and ACTA2 shRNA-transfected cells were resuspended in culture media (5 × 10^4^ cells/well) and added to the matrigel-coated upper compartment of invasion chambers. Fresh culture media with 5% FBS was added to the lower compartment. After 48 h, cells on the upper side of the filter were removed using cotton swabs. The underside of the filter was fixed in 100% methanol, washed in PBS, and stained using hematoxylin and eosin (H&E). Cells that invaded the matrigel were analyzed using a Scanscope XT apparatus (Aperio Technologies, Vista, CA, USA).

### Measurement of metastatic potential and immunohistochemistry using orthotopic xenograft models

To establish a nude mice xenograft model, we used 6- to 8-week-old female Balb/c nude mice (Orient Bio, Korea), weighing about 18–22 g. Mice were kept in pathogen-free animal housing in accordance with the National Institutes of Health Guide for the Care and Use of Laboratory Animals. Empty and ACTA2 shRNA-transfected 4T1 mammary carcinoma cells (1 × 10^5^ cells/100 μl) were directly injected into the right secondary mammary fat pad (n = 5/group). Tumor sizes in empty and ACTA2 knockdown mice were measured using digital calipers. Volume was measured using the formula: V=1/2 length × (width)^2^. Tumor sizes at primary sites were calculated using average relative tumor volume in each group (empty or ACTA2-knockdown).

After 4 weeks, lung tissues from empty and ACTA2 knockdown mice were fixed with formalin and embedded in paraffin. Tissue sections were cut and deparafinized in xylene, dehydrated in graded alcohol and hydrated in water. Tissue sections (4 μm) were immunohistochemically stained using H&E, proliferating cell nuclear antigen (PCNA), and ACTA2. Slides were analyzed using a Scanscope XT apparatus (Aperio Technologies, CA, USA).

### Statistical analysis

Statistical significance was determined using Student's *t*-test. Results were presented as means ± SEM. All *p*-values were two-tailed and differences were considered statistically significant when *p* < 0.05. Statistical analysis was performed using Microsoft Excel (Redmond, WA, USA).

## SUPPLEMENTARY MATERIALS FIGURES


