# Supplementary material for: Dimerization of EGFR and HER2 induces breast cancer cell motility through STAT1-dependent ACTA2 induction
Source: Oncotarget. 2016 Jul 26;8(31):50570–81. doi: 10.18632/oncotarget.10843 (PMC5584169; doi:10.18632/oncotarget.10843)
Supplement: Supplementary file 1 [file oncotarget-08-50570-s001.pdf]

Dimerization of EGFR and HER2 induces breast cancer cell motility through STAT1-dependent ACTA2 induction

SUPPLEMENTARY FIGURES

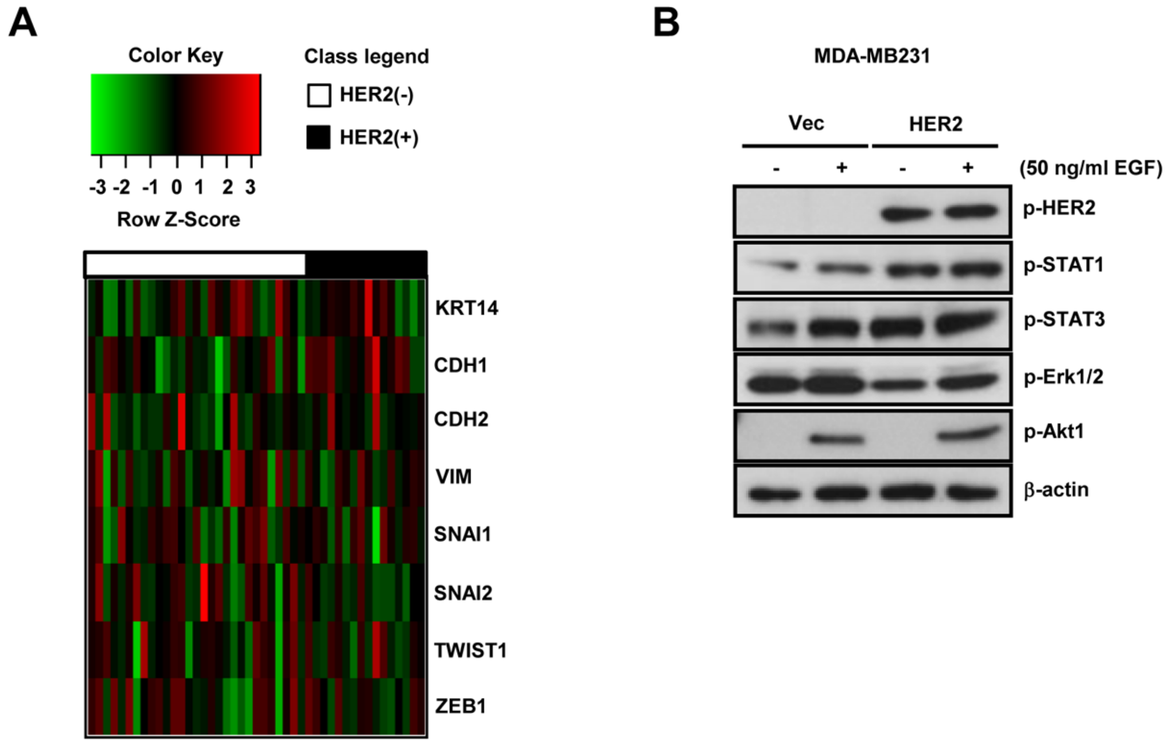

Supplementary Figure S1: A. Heatmap of KRT14, CDH1, CDH2, VIM, SNAI1, SNAI2, TWIST1, and ZEB1 expression in tumors from breast cancer patients generated using R statistical software. B. After serum starvation for 24 h, empty and HER2-overexpressing MDA-MB231 cells were treated with 50 ng/ml EGF for 24 h. The levels of p-HER2 (pT877), p-STAT1 (pS727), p-STAT3 (pY705), p-Erk1/2 (pT202/pT185), p-Akt1 (pS473), and β-actin were analyzed by western blots. Results are representative of three independent experiments.

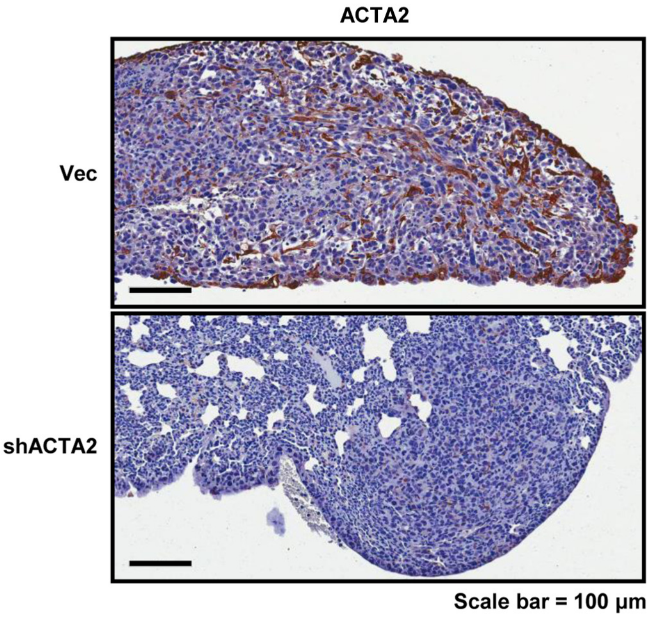

Supplementary Figure S2: The levels of ACTA2 expression in lung metastatic tumors were analyzed by immunohistochemistry.

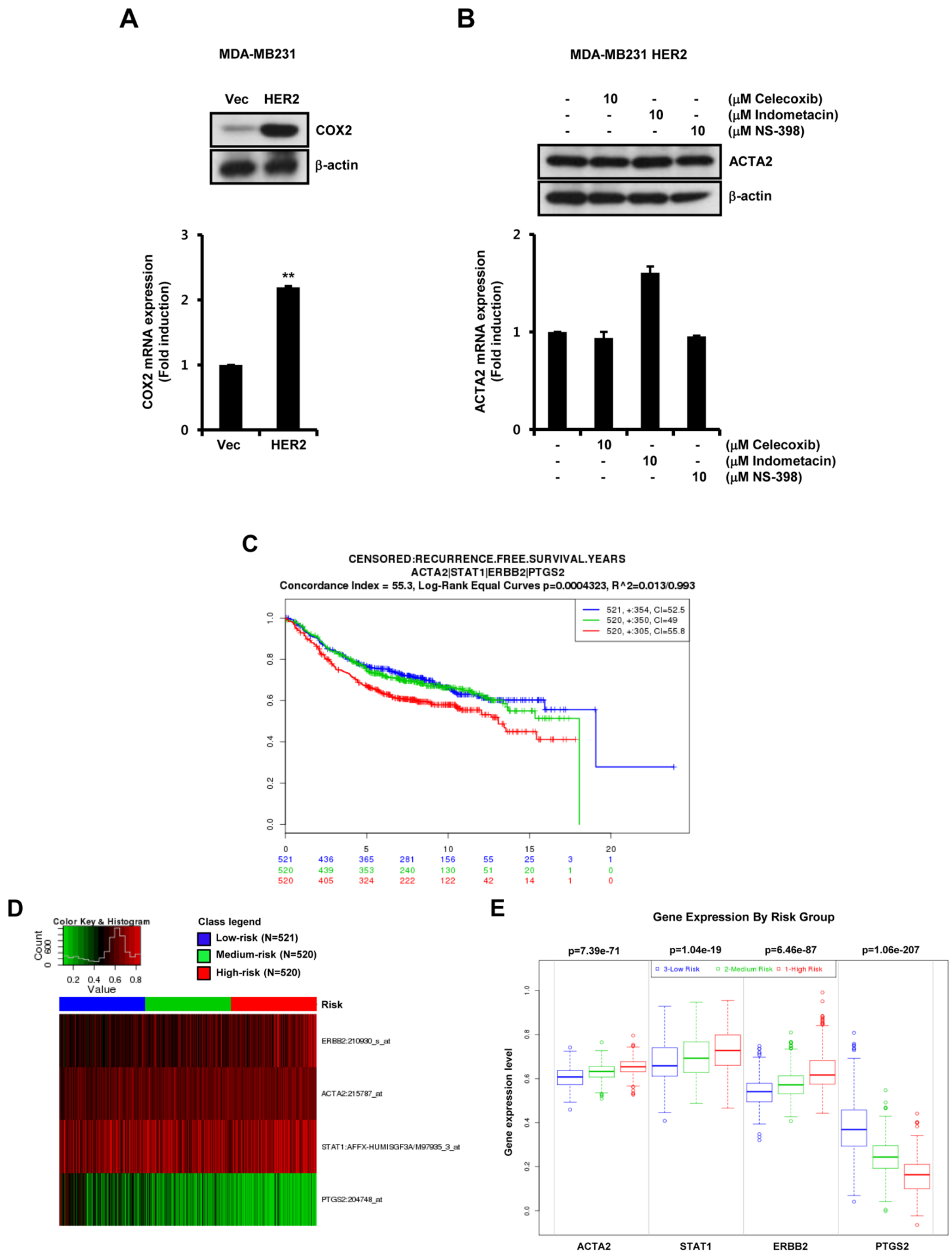

Supplementary Figure S3: A. COX2 and β-actin expression analyzed by western blots. COX2 mRNA expression was analyzed by real-time PCR. **B.** After serum starvation for 24 h, HER2-overexpressing MDA-MB231 cells were treated with 10 μM celecoxib, indometacin, or NS-398 for 24 h. The levels of ACTA2 and β-actin expression were analyzed by western blots. ACTA2 mRNA was analyzed by real-time PCR. Results are representative of three independent experiments. Values are means ± SEM. ** $p < 0.01$ vs. Vec. Vec; empty vector. **C-E.** The clinical value of ACTA2, STAT1, HER2, and COX2 was analyzed by SurvExpress software. (C) Kaplan-Meier curves (recurrence-free survival). (D) Heatmap. (E) Box plots.
